# The impact of microbiome dysbiosis on T cell function within the tumor microenvironment (TME)

**DOI:** 10.3389/fcell.2023.1141215

**Published:** 2023-03-17

**Authors:** Michelle P. DiPalma, Joseph N. Blattman

**Affiliations:** ^1^ School of Life Sciences, Arizona State University, Tempe, AZ, United States; ^2^ Biodesign Institute, Center for Immunotherapy, Vaccines and Virotherapy (CIVV), Arizona State University, Tempe, AZ, United States

**Keywords:** microbiome, T cell, tumor microenvironment (TME), dysbiosis, metabolites, T cell signaling, short chain fatty acids (SCFAs), immunotherapy

## Abstract

Insights into the effect of the microbiome’s composition on immune cell function have recently been discerned and further characterized. Microbiome dysbiosis can result in functional alterations across immune cells, including those required for innate and adaptive immune responses to malignancies and immunotherapy treatment. Dysbiosis can yield changes in or elimination of metabolite secretions, such as short-chain fatty acids (SCFAs), from certain bacterial species that are believed to impact proper immune cell function. Such alterations within the tumor microenvironment (TME) can significantly affect T cell function and survival necessary for eliminating cancerous cells. Understanding these effects is essential to improve the immune system’s ability to fight malignancies and the subsequent efficacy of immunotherapies that rely on T cells. In this review, we assess typical T cell response to malignancies, classify the known impact of the microbiome and particular metabolites on T cells, discuss how dysbiosis can affect their function in the TME then further describe the impact of the microbiome on T cell-based immunotherapy treatment, with an emphasis on recent developments in the field. Understanding the impact of dysbiosis on T cell function within the TME can carry substantial implications for the design of immunotherapy treatments and further our understanding of factors that could impact how the immune system combats malignancies.

## Introduction

Microbial species inhabit nearly every organ of the human body; their significance has recently been established in proper health and immune cell function, with potential impacts in the tumor microenvironment (TME) through the presence or absence of microbial-derived metabolites such as short-chain fatty acids (SCFAs), that can impact T cell functioning. The human microbiome comprises a complex network of various organisms, including those of bacterial, archaeal, fungal, viral, and protozoan populations, many of which are capable of symbiotic or pathogenic manifestations on the host, particularly when oscillations in microbial composition occur (Riiser, 2015). In a healthy host, microbial populations typically outnumber human cells; current studies have estimated the number of bacteria alone in the human body is roughly the same as that of human cells (Sender et al., 2016). Technological advancements such as metagenomic sequencing and sophisticated data analysis have allowed scientists to characterize the abundance and diversity of the human microbiome, classifications that have allowed researchers to elucidate the potential mechanisms by which these species impact health and disease (Freilich et al., 2009; Qin et al., 2010; Methé et al., 2012). Studies support the necessity of a diverse, stable, and balanced microbiome to maintain general health and proper immunity to disease, with negative impacts during microbiome dysbiosis (Tuddenham and Sears, 2015). Microbial dysbiosis is an imbalance in the composition of microbial communities within a host resulting in perturbations from normal cellular and organ function, illness, or reduced treatment efficacy for an infection or disease (Petersen and Round, 2014). The etiology of dysbiosis is diverse and includes pathologies resulting from inflammation, infection, diet, genetics, and antibiotic administration (Willing et al., 2010; Claesson et al., 2012; Pham and Lawley, 2014). Current studies have also demonstrated the impact of host-microbiome interactions during dysbiosis on cancer progression and treatment, including disease presence and chronic inflammation (Czesnikiewicz-Guzik and Müller, 2018). Dysbiosis is believed to impact the regular functioning of immune cells caused by modifications in microbial-produced metabolites needed for proper performance (Arpaia et al., 2013; Luu et al., 2019). Such alterations in immune cells can impact not only how we respond to pathogenic infections but also the immune response to neoplastic events.

The degree of T cell tumor site infiltration and proper function within tumor sites can significantly affect tumor progression or regression (Al-Shibli et al., 2008). Therefore, an alteration in T cell function can cause a massive change in the efficacy of how T cells respond within the TME and, ultimately, their ability to clear malignancies. Understanding the mechanistic impact of microbial composition on T cells and their function within the TME is essential if we hope to understand and improve treatment strategies for malignancies. Here, we review canonical T-cell responses to malignancies, the impacts of the microbiome in the context of its typical and dysbiotic state on T-cell signaling and further how this can change T-cell function within the TME, recent discoveries in the field, and the potential that research stemming from these investigations can have on how we design and administer cancer therapies.

## Conventional anti-tumoral activity of T-cells

Adaptive immune cells, such as T cells, play a considerable role in the antitumor immune response, with the presence of tumor-infiltrating T lymphocytes exhibiting positive prognostic value across a wide range of cancers (Zhang et al., 2003; Taylor et al., 2007). To understand how microbiome dysbiosis can affect the ability of T cells to react to malignancies, we must first understand conventional anti-tumoral T-cell mechanisms.

### Typical CD4 T cell signaling and function in response to malignancies

**FIGURE 1:**
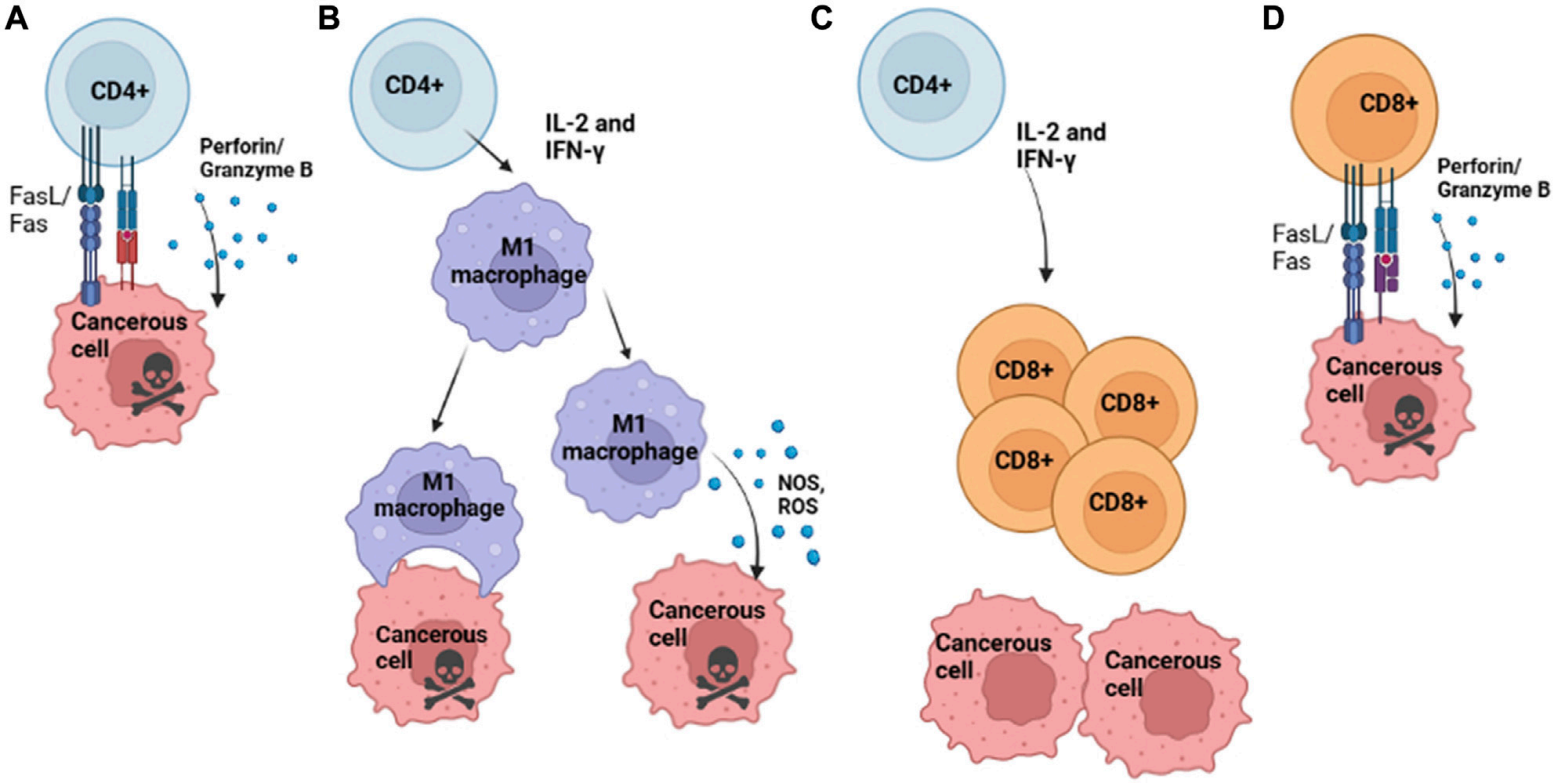
Conventional Mechanisms of T Cell Anti-Tumoral Activity. **(A)** Th1 differentiated CD4 T cell killing of cancerous cells that present neoepitopes on MHC II via secretion of perforin and granzyme B. **(B)** CD4 T cells release cytokines IL-2 and IFN-γ to recruit M1 macrophages; activated M1 Macrophages can kill cancerous cells either by direct phagocytosis or release of NOS or ROS, which results in tumor cell death and continue to secrete IL-12, among other cytokines which recruit T cells to the site of the tumor. **(C)** Activated CD4 T cell release of cytokines such as IL-2 and IFN-γ, which recruits CD8 T cells to the site of tumor growth. **(D)** CD8 T cells directly kill cancerous cells that present neoepitopes via MHC I via the Fas/FasL and the release of perforin/ granzyme B, resulting in tumor cell killing. Image Created with BioRender.com.

CD4 T cells are directly and peripherally involved in the antitumor immune response through effects on innate and adaptive immune cells such as macrophages and T cells. Individually, CD4 T cells can be cytotoxic to incipient and progressive malignancies, acting directly on unhealthy cells that present neoepitopes to eliminate them through differentiation into Th1 cells and ensuing perforin/granzyme B-dependent killing or target cell elimination *via* ligation of Fas/FasL (or other TNF/TNFR family) death receptors (Figure 1A) (Lundin et al., 2004). Peripherally, CD4 T cells can also secrete IL-2 and IFN-γ, which have been found to elicit M1 macrophages to inhibit tumor propagation *via* secretion of nitric oxide synthase (iNOS), reactive oxygen species (ROS), and IL-12, resulting in direct and indirect clearance of tumor cells. Additionally, M1 macrophages can engulf malignant cells to eliminate them (Figure 1B) (Zhou et al., 2021). These M1 mechanisms are productive during MHC class II negativity, which can result from the loss of MHC Class II trans-activator (CIITA) expression during carcinogenesis (Haabeth et al., 2014). Additionally, CD4 T cells are necessary to support continued CD8 memory T cell survival and function (Janssen et al., 2003). They secrete cytokines, such as INFγ and IL-2, that can act on CD8 T cells to improve the anticancer immune response (Figure 1C) (Ossendorp et al., 1998).

### Typical CD8 T Cell signaling and function in response to malignancies

The presence of CD8 T cells within the TME has been associated with improved tumor clearance and overall prognosis, often characterized by the concomitant presence of pro-inflammatory cytokines such as Type I IFN (Trujillo et al., 2018). The mechanisms by which CD8 T cells within the TME are believed to eliminate cancerous cells are *via* ligation of the Fas ligand (Fas L) on T cells with the Fas receptor (FasR) on target cells or by the perforin/granzyme B pathway, with a preference for FasL/FasR pathway for cancerous cells (Figure 1D) (Chávez-Galán et al., 2009). Though these mechanisms can aid in the clearance of tumors, tumor-infiltrating lymphocytes often display upregulation of inhibitory markers, such as PD-1 and CTLA-4, which bind PD-L1 and CD80/CD86, respectively; these can halt anti-tumoral effector functions and result in reduced effector cytokine production (Ahmadzadeh et al., 2009).

## Molecular impact of the microbiome on anti-tumoral activity of T-cells

There is evidence that microbial species within the mucosal tissue of a host and within the tumor microbiome contribute to patient tumor immune response variations, with correlations between metabolic functions of microbes present within the TME and clinical patient presentation (Nejman et al., 2020). These trends have been documented for several pancreatic, bone, and breast cancers (Riquelme et al., 2019; Nejman et al., 2020; Banerjee et al., 2021). Alteration of the host microbiome can change host cell function, including that of innate and adaptive immune cells (Russo et al., 2016; Thaiss et al., 2016). The mechanisms underlying these cellular changes are still being investigated but have been better characterized in recent studies and are believed to be primarily associated with alterations in metabolite secretions by microbial species that subsequently impact immune cell function (Luu et al., 2019; Yang et al., 2020). In particular, microbes produce fermentation products known as short-chain fatty acids (SCFAs); these free fatty acids contain short aliphatic carbon chains and are composed of less than six carbons. Typically when referring to SCFAs, the following are included: formic acid (C1), acetic acid (C2), propionic acid (C3), butyric acid (C4), and valeric acid (C5) (Tan et al., 2014). SCFAs are predominantly water-soluble, and therefore easily transported throughout the body, and are believed to play a significant role in the differentiation, function, and regulation of T cells and other immune cells through the promotion of pro- or anti-inflammatory cytokines needed for particular effector functions (Arpaia et al., 2013; Ryu et al., 2022). Thus, alterations in microbiome composition and consequent changes in microbial metabolite secretion may disrupt T cells’ conventional effector functions against malignancies.

### Tumor-specific microbiome associations with malignancies

Microbial composition within the TME varies across cancers and further differs from adjacent healthy tissues, even in solid tumors with no direct contact with the external environment (Nejman et al., 2020). Several cancers, including breast, lung, ovarian, colorectal, melanoma, brain, prostate, and bone, have exhibited the presence of specific microbial species contributing to a dysbiotic state within tumor tissue (Sfanos et al., 2008; Apostolou et al., 2011; Castellarin et al., 2012; Thompson et al., 2017; Costantini et al., 2018; Greathouse et al., 2018; Nejman et al., 2020). These tumor-specific microbial populations can sometimes vary for different cancer types within the same organ systems (Banerjee et al., 2018). Considering the known impact of microbial changes on immune cell function, changes within the TME regarding tissue-specific microbial populations may initiate changes in T cell function within neoplasms (Nejman et al., 2020). Intriguingly, *Rotter-Maskowitz et al.* recently identified correlations between intratumoral bacterial presence and predicted clinical presentation and response to anticancer treatment (Nejman et al., 2020). Similar concepts can be traced back to William Coley in the late 19th century, who showed that injecting killed bacterial species into tumor tissue resulted in tumor regression, which we now believe is due to adjuvant effects *via* activation of innate immune receptors and engagement of subsequent immune responses within the tumor (Kopenhaver et al., 2020). Further investigations are needed to determine if bacterial presence in cancer impacts cancer progression *via* changes in immune cell function or if metabolites preferred by certain bacteria that are present as a result of cancer progression provide a niche for those found in specific tumor tissues, the presence of particular bacterial species in tumor tissue compared to healthy tissue indicates a potential avenue for understanding better what factors impact cancer progression within the TME (Thompson et al., 2017).

### Gut microbiome associations with malignancies

Investigations during cancer progression and treatment suggest certain gut microbial presence outside the TME correlate with systemic inflammatory processes that affect the TME, including the upregulation of pro-inflammatory cytokines such as tumor necrosis factor (TNF) caused by increased immune cell responses, resulting in more significant tumor regression (Iida et al., 2013). These systemic alterations could be associated with secreted metabolites from specific bacteria, noting that metagenomic studies concluded that the enrichment of anabolic pathways resulting from cellular metabolism and differences in pro-inflammatory cytokines caused by some bacteria’s presence affect tumor response (Gopalakrishnan et al., 2018). Additionally, several studies suggest that gut microbiome composition plays a role in cancer progression at mucosal sites and in tumors not confined to mucosal tissue, which may be partly due to metabolites produced by microbes in the gut (Zhuang et al., 2019; Sánchez-Alcoholado et al., 2020).

### Transport of microbial-derived metabolites to T immune cells

Metabolites can be produced by gut-specific or organ-specific bacteria and passively or actively transported to other locations impacting organ and cellular function at local and peripheral sites (Cummings et al., 1987; Kamp and Hamilton, 2006). For this reason changes in microbiome composition will also impact metabolite presence. For instance, during dysbioisis, certain SCFAs or other metabolites may be reduced in quantity if the bacteria that produce them are no longer present. Active diffusion of metabolites in T cells can occur *via* membrane transporters, including MCT1 (monocarboxylate transporter-1/Slc16a1) and SMCT1 (sodium-coupled monocarboxylate transporter-1/Slc5 a8) (Park et al., 2014). Once inside the cell, SCFAs are known to act *via* G-protein-coupled receptor (GPCR) signaling, inhibition of histone deacetylase (HDAC), production of acetyl-CoA, and further changes in the metabolism of the cell resulting in increased or decreased functionality (Kim, 2021). Evidence shows that SCFA presence activates mTOR, and STAT3 in T cells *via* GPCR41, GPCR43, GPCR109a resulting in Blimp-1 expression, which triggers the expression of many downstream signaling cascades (Zhao et al., 2018). Studies in germ-free mice suggest that changes in microbiota can directly impact the expression of toll-like receptors (TLRs) (Lundin et al., 2008) and can affect antigen-presenting cell presence, T cell differentiation, and systemic immunity (Rangan and Mondino, 2022).

### Molecular effects of dysbiosis on CD4 T cell signaling and function

**FIGURE 2:**
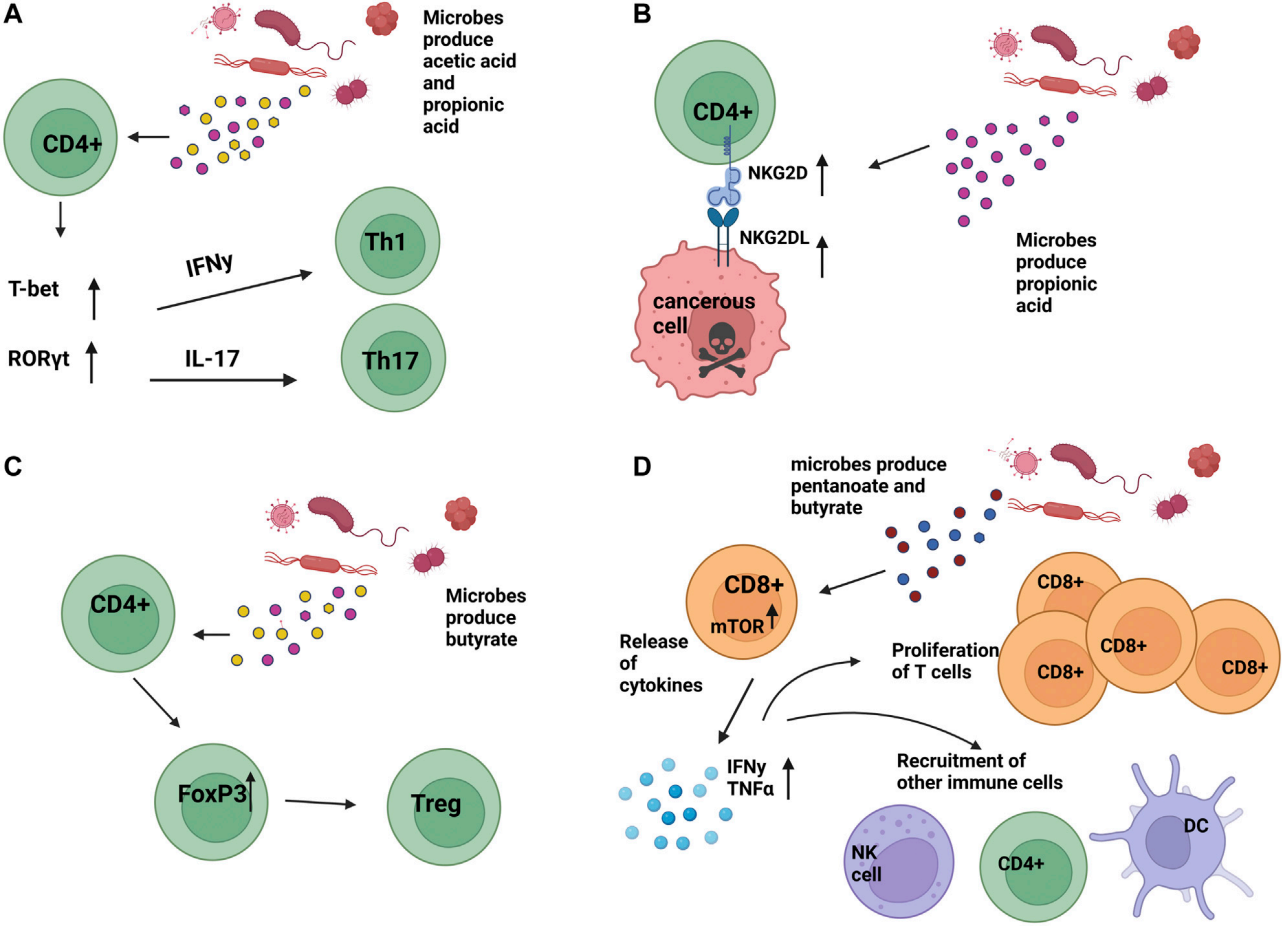
Impact of microbial secreted metabolites on T cell function **(A)**. Bacterial-produced acetic acid (C2) and propionic acid (C3) can result in upregulation of T-bet and RORγt, causing upregulation of IFNy and IL-17 and subsequent differentiation into Th1 and Th17 T cells, respectively. **(B)**. Microbial produced propionic acid (C3) has resulted in the upregulation of NKG2D and NKG2DL on CD4 T cells, CD8 T cells, and NK cells, contributing to an anti-tumoral environment and death of cancer cells. **(C)**. Microbial-produced butyrate has been shown to cause the differentiation of T-regulatory cells (Tregs) in a concentration-dependent manner. **(D)**. Microbial-produced pentanoate and butyrate have been shown to contribute to increased CD8 T cell effector functions. *Image created with* *BioRender.com* .

Recent studies showed C2, C3, and C4 metabolites, which can be produced by microbiota, could exhibit immunomodulatory functions by altering CD4 T cell differentiation in a concentration-dependent manner, as high concentrations of C2 and C3 drove expression of IL-17A, IL-17F, RORα, RORγt, T-bet, and IFN-γ, cytokines associated with Th17 and Th1 profiles (Figure 2A). (Park et al., 2014). More specifically, the natural killer group 2, member D (NKG2D) ligand system is a central immunomodulatory system in which immune cells recognize NKG2DL on stressed or infected cells through NKG2DR, present on immune cells such as CD4 T cells, CD8 T cells, and NK cells, activating effector functions (Groh et al., 2003). C3 produced by *propionibacteria* during cellular metabolism can induce the expression of NKG2D ligands MICA/B on both activated T lymphocytes and cancer cells in an intracellular calcium-dependent manner, allowing for proper immune effector function and potential prophylaxis of malignant cells (Figure 2B). (Andresen et al., 2009). This finding also implies the absence of C3 during the elimination of microbial populations that produce it can limit the degree of NKG2D ligands and, therefore, limit subsequent NKG2D/NKG2D interactions, resulting in a reduced overall T cell effector function within the TME. Further, C4 presence was shown to drive Treg differentiation in a concentration-dependent manner both *in vitro* and *in vivo* (Figure 2C). (Furusawa et al., 2013; Kespohl et al., 2017). Additional studies have indicated issues with the development of regulatory T cells (Tregs) in antibiotic-treated mice, indicating a requirement for healthy intestinal microbiota to achieve even normal Treg development (Han et al., 2021).

### Molecular impact of dysbiosis on CD8 T cell signaling and function

In pre-clinical mouse models, microbial dysbiosis induced by maternal antibiotic treatment found that the offspring had altered CD8^+^ T Cell receptor signaling apparent by an inability to sustain interferon-gamma (IFN-γ) production *in vivo* after vaccination and *in vitro* upon T cell receptor (TCR) stimulation. Resultantly, these cells did not maintain protein tyrosine phosphorylation and Erk1/2 activation, which are necessary for the proper functioning of CD8^+^ T Cells (Brownlie and Zamoyska, 2013). Further, SCFA presence was shown to enhance the function of cytotoxic T lymphocytes through an increased function of mTOR post-treatment of T cells with pentanoate and C4 and driving supplementary inhibition of class I histone deacetylase activity. mTOR typically drives differentiation into Th1, Th2, and Th17 but is also a critical regulator in CD8 T cell differentiation through the regulation of cytolytic effector molecules (Finlay et al., 2012; Liu et al., 2015). Effector molecules such as CD25, IFN-γ, and TNF-α were then elevated in these treated cells, demonstrating enhanced cytotoxic activity and potential for pentanoate and C4 as supplements to cancer treatment for some malignancies (Figure 2D). (Schiweck et al., 2022).

### Elimination of microbial species by antibiotics and the resulting effect on T cell populations

Considering the impact of microbial species on T-cell function, clinicians should be cognizant of the immunopharmacological behavior of antibiotics regarding microbial species-specific elimination based on antibiotic type. For instance, neomycin, which predominantly eradicates facultative gram-negative species, and vancomycin which predominantly eradicates gram-positive species, have both been associated with the reduced expansion of T cells in mouse models during antibiotic administration (Duan et al., 2010; Cheng et al., 2017). Cocktails of ampicillin, vancomycin, neomycin and metronidazole (AVNM) have been associated with lower immune function and decreased concentrations of bacterial metabolites C3 and C4 in mice (Ubeda and Pamer, 2012). γδ T cells are a part of the Th17 subset and are a source of the pro-inflammatory cytokine IL-17. Antibiotics have also been shown to modulate γδ T cell populations, with variations depending on the antibiotic type used and species of bacteria eliminated (Duan et al., 2010). Understanding which antibiotics eliminate microbial populations may be critical when designing patient treatment regimens to reduce the chances of anomalous immune cell function.

## Interactions of dysbiosis on T cell-based immunotherapy in the TME

Both CD4 and CD8 T cells play an essential role in the clearance of malignancies. The impact of microbiome dysbiosis on immune cell function generates a challenging dilemma, considering that many immunotherapies in pre-clinical and clinical use, such as immune checkpoint blockade and adoptive cellular therapies, are T-cell-based.

### Impact of dysbiosis on T cell dependent immune checkpoint blockade

It has been documented that specific microbes in the gut can impact ICB immunotherapy approaches across several cancers (McCulloch et al., 2022). For instance, anti-CTLA-4 treatment for melanoma relies on the presence of *Bacteroides* species; additionally, the treatment showed no effect on germ-free and antibiotic-treated mice (Vétizou et al., 2015). *Bacteroides* species can be propionogenic, having the capacity to generate the SCFA C3. Therefore, the absence of C3 can alter the proper function of T cells, which anti-CTLA-4 treatment relies on (Louis et al., 2014). In a separate study, the introduction and restoration of propionogenic bacteria during antibiotic-induced dysbiosis resulted in the restoration of C3 levels, indicating that the re-establishment of propionogenic bacteria could counteract decreases in SCFAs required for the efficacy of particular immunotherapy (El Hage et al., 2019). In mice and humans, high C4 concentrations in the blood were associated with resistance to anti-CTLA-4 therapies, evidenced by restrained upregulation of B7 on T cells (Coutzac et al., 2020). Similar results of reliance of immunotherapy efficacy on bacterial presence were seen in anti-PD-L1 treatment for melanoma, which depended on the presence of *Bifidobacterium* (Sivan et al., 2015). *Bifidobacterium* species produce SCFAs C2, C3, and C4 SCFAs that contribute to immune cell function (Louis et al., 2014). Strikingly, more recent investigations have shown fecal transplants from ICB responders to non-responders for melanoma treatment saw that more than one-third of human patients previously unresponsive to treatment become responsive after transplants (Davar et al., 2021).

### Impact of dysbiosis on adoptive T-cell therapy

Recent studies in mice have further shown that differences in gut microbiome composition and dysbiosis due to antibiotic administration could alter the efficacy of adoptive T-cell cancer treatments. However, these changes in response to treatment are likely species-specific since they vary based on the type of antibiotic administered. In fact, some mice treated with vancomycin displayed an increase in CD8α+ dendritic cells (DCs) with supplemental decreases in tumor burden in an IL-12-dependent manner. At the same time, alternative antibiotics did not produce the same effect (Uribe-Herranz et al., 2018). Additional studies have indicated that severe cytokine release syndrome during CAR- T cell therapy is associated with particular microbiome alterations and a higher abundance of *Bifidobacterium*, *Leuconostoc*, *Stenotrophomonas*, and *Staphylococcus* and that desired responses may require specific gut microbial presence (Hu et al., 2022; Smith et al., 2022). These results demonstrate that microbiome composition can also impact T-cell therapies whose efficacy relies on proper immune cell function.

### The impact of diet on T cell based cancer therapies

It is well known that diet can influence the establishment of microbial communities within a host (David et al., 2013). Since we now know that microbial community composition can impact T cell function, investigations regarding how diet can impact cancer treatments that rely on T cell function have been recently investigated. In mice, the western diet of high fat, high carbohydrate, and low fiber diet can decrease downstream production of short-chain fatty acids (SCFA), which originate from microbiota; this could impact T cell function and efficacy of treatments that rely on T cells. (Statovci et al., 2017). Other findings have shown that ketogenic diets can increase antitumor immunosurveillance by reducing PD-L1 expression on tumor cells in a malignant glioma model. (Lussier et al., 2016). Additionally, when placed on a ketogenic diet, mice have displayed enhancement of the anticancer effects of PD-1 blockade. (Ferrere et al., 2021). More recent studies have shown that when fucoidan, a polysaccharide naturally derived from brown algae, was co-administered with ICB treatment, it significantly improved the antitumoral activity of PD-1 antibodies in a murine melanoma model *in vivo* through consistent activation of tumor-infiltrating CD8^+^ T cells. (Yang et al., 2021). Recent studies also indicate that calorie restriction can increase the antitumoral ability of T cells (Pietrocola and Kroemer, 2019), an approach that, when applied to a murine triple-negative breast cancer model, augmented radiation efficacy (Saleh et al., 2013). Further, clinical trials analyzing melanoma patients showed that patients who consumed a high-fiber diet were five times more likely to respond to PD-1 therapy (Spencer et al., 2019). Modulation of diet serves as a potential interventional strategy that can be used to enhance T cell-based immunotherapies therapies in the future.

## Discussion

With the onset of technological advancements such as molecular sequencing and sophisticated Metabolic-network modeling, insights into how microbiome composition impacts health, disease, immune cell function disease, cancer, and immune cell function have been recently better illuminated. Further insights into cohesion between microbiome composition and proper immune cell function may be a helpful resource that will eventually allow scientists to regulate the immune response to and clearance of malignancies. Further, broadening our understanding of T cell function in the TME concerning microbiome composition may improve our understanding of how best to administer current antineoplastic drugs and therapies.

There also exists potential to exploit the dysbiotic microbiome’s impact on immune cell function as an augmentation to anti-tumoral therapies for some cancers originating in common lymphoid progenitors (CLP). For instance, patients with cutaneous T-cell lymphoma (CTCL) reported decreased overall tumor burden when treated with an aggressive antibiotic regimen. Upon immunohistochemistry analysis, samples displayed a decrease in interleukin-2 high-affinity receptors in T cells at these sites, indicating a decline in mechanisms that allow for T cell proliferation (Lindahl et al., 2019). Similar regimens have also been pursued in Mucosal Associated Lymphoid Tissue (MALT) lymphomas, improving 5- year survival rates (Ferreri et al., 2018). These incidents demonstrate the importance of identifying how antibiotics can affect immune cells and the specific malignant cells clinicians might target during treatment.

Though the composition of the microbiome and microbial dysbiosis can impact immune cell function and subsequent immune response to malignancy, research gaps still exist that must be pursued to mitigate the adverse effects of microbial dysbiosis and immune system dysfunction. Further research is needed into what comprises the “optimal” microbiome and whether this composition differs for particular diseases and malignancies. Current data indicate a requirement for the presence of specific microbial species’ therapeutic efficiency (Sivan et al., 2015), but this also tends to vary from cancer to cancer, making it difficult to classify what an optimal microbiome for patients might look like. For this reason, a more significant effort is needed to determine what microbes may offer benefits during cancer treatment and what causes these microbiomes to be beneficial or harmful in particular organ systems. Since both gut and tumor-specific microbial composition can potentially permute disease progression, identifying what comprises microbial populations both locally and systemically across specific cancers may serve as a helpful resource for future diagnostic approaches. Several microbial signatures between blood and tissues have already been identified across cancers, indicating a potential diagnostic approach to cancer treatment that may soon be available (Poore et al., 2020).

Further research into how the dysbiotic state of the microbiome can impact T cell function can lead to potentially better treatments for cancer patients through the modulation of microbes present in the host. Knowledge pertaining to which bacterial communities and their associated mechanisms are needed for proper immune cell function would allow physicians to be aware of the implications associated with specific antibiotic use and subsequent species-specific elimination of bacterial communities in conjunction with ICB treatments. Overall, expanding our knowledge about the microbiomes’ interconnection with the immune system and T-cell function during cancer progression and treatment can improve our knowledge of how to design best and administer cancer treatments and ultimately improve patient outcomes.
